# Mothers’ Insistence when Prohibiting Infants from Harming Others in Everyday Interactions

**DOI:** 10.3389/fpsyg.2016.01448

**Published:** 2016-10-11

**Authors:** Audun Dahl

**Affiliations:** Department of Psychology, University of California, Santa CruzSanta Cruz, CA, USA

**Keywords:** moral development, mother–infant interactions, social domain theory, transgressions, naturalistic interactions

## Abstract

Social interactions about transgressions provide a context for the development of children’s moral aversion to harming others. This study investigated mothers’ insistence when communicating the prohibition against harming others to infants in everyday home interactions. Mothers’ reactions to infants’ use of force against others (*moral harm* transgressions) were compared to their reactions to transgressions pertaining to infant wellbeing (*prudential*) and transgressions pertaining to inconvenience (*pragmatic*). Twenty-six infants and their families participated in 2.5-h naturalistic home observations when infants were 14, 19, and 24 months old. Mothers’ interventions on moral harm transgressions involved increased use of physical interventions and direct commands, and decreased use of distractions, softening interventions, and relenting/compromising, compared to their interventions on prudential and pragmatic transgressions. Children showed the greatest immediate compliance with, and least protests against, maternal interventions on moral harm transgressions.

## Introduction

When infants hit, bite, or kick they elicit reactions that set the stage for their development of a moral aversion to harming others. Rates of harmful behaviors increase in the first half of the second year and decline over the subsequent years (Hay, 2005). By 3 years, children judge harming others as morally wrong (Smetana and Braeges, 1990; Nucci and Weber, 1995; Dahl and Kim, 2014). Most researchers agree that infants’ everyday experiences with transgressions contribute to the development of moral and other norms, but have different hypotheses about what those experiences are (Turiel, 1983; Grusec, 2011; Bloom, 2013; Smetana, 2013). This study investigated a novel dimension of infants’ experiences with transgressions that may convey the unique importance of the moral prohibition against harm: Variability in mothers’ behavioral insistence on everyday rules.

“Insistence” here refers to mothers’ tendency to explicitly convey that the child is doing something wrong. Being insistent means, for instance, physically holding the child back, saying “no,” or refusing to relent or compromise. In contrast, distracting the child, comforting the child, or compromising indicates a lack of insistence, in this definition. Insistence is a behavioral reflection of how important it is for a caregiver to communicate a given prohibition to a child. Insofar as children perceive caregivers to be more insistent on some prohibitions than others, children may come to perceive some prohibitions as more important to parents than others. This perception may in turn affect children’s understanding of the nature of these events and their tendency to accept or challenge parents’ prohibitions in future interactions.

Caregiver insistence on the moral prohibition against harming others may be especially relevant in the second year. The second year is characterized by both limited linguistic abilities and an increase in parent–child conflicts, including conflicts about infant use of force against others (Rijt-Plooij and Plooij, 1993; Fenson et al., 1994; Biringen et al., 1995; Hay, 2005). The co-occurrence of limited channels of communication and high frequencies of transgressions may lead caregivers to prioritize which prohibitions to insist upon (or to “pick one’s battles,” as one participating mother said).

This study investigated the hypothesis that mothers in U.S. middle class families would, on average, be more insistent on the prohibition against harming others than on other types of prohibitions during the second year of life. The study focused on mothers because infants commonly spend a substantial amount of their time with their mothers, but this does not imply that other social agents are unimportant for social development. Mothers’ reactions to interpersonal harm (e.g., hitting or biting, termed *moral harm* transgressions) were compared to mothers’ reactions to *prudential* transgressions (pertaining to the child’s welfare, e.g., climbing on a couch or moving toward a staircase) and *pragmatic* transgressions (pertaining to inconvenience, e.g., spilling or creating disorder) (Tisak, 1993; Gray et al., 2012; Dahl and Campos, 2013; Dahl and Kim, 2014).^1^

A second novel feature of the study was to investigate of mothers’ reactions to prudential and pragmatic transgressions during the second year of life. Although transgressions relating to the child’s wellbeing (prudential) and the creation of mess and other inconvenience (pragmatic) are common occurrences during infancy, caregiver reactions to such transgressions in infancy have previously only been investigated using maternal report (Smetana et al., 2000; Dahl and Campos, 2013).

Several theorists have proposed those children’s construct moral, prudential, and pragmatic concepts from everyday social experiences (Turiel, 1983, 2015; Smetana, 2013). A key corollary of this proposition is that children have different types of experiences with moral harm, prudential, and pragmatic transgressions. For instance, caregivers’ heightened insistence on the prohibition against harming others could help children view harming others both as wrong and as distinct from other types of transgressions (Tisak, 1993; Nucci and Weber, 1995; Smetana et al., 2012; Smetana, 2013; Dahl and Kim, 2014; Turiel, 2015). A first, high levels of insistence unequivocally conveys to children that they are doing something considered wrong. Moreover, heightened insistence on moral harm prohibitions relative to other prohibitions may help children recognize how moral harm transgressions differ from other types of transgressions (Dahl et al., 2011; Dahl and Campos, 2013; Dahl and Tran, 2016). At least by 3-to 4 years of age, children have come to see that the moral prohibition against harm has a different justification than pragmatic, prudential, and conventional norms, is not alterable by consensus, and should be applied universally (Smetana and Braeges, 1990; Tisak, 1993; Nucci and Weber, 1995; Smetana et al., 2012; Dahl and Kim, 2014).

Several theorists have argued for the importance of considering the nature of children’s transgressions when studying parental reactions and their influence on children (Grusec and Goodnow, 1994; Holden and Miller, 1999; Turiel, 2005; Grusec and Davidov, 2010). However, much of the research on how parents respond to infant transgressions focus on reactions to a single transgression type, such as parental attempts to keep infants from approaching a prohibited toy in a research laboratory (e.g., LeCuyer-Maus and Houck, 2002; Turiel, 2005; Kochanska et al., 2007). Without questioning the importance of the prohibited toy paradigm, the present research investigated a wider variety of transgression types encountered by infants and their mothers in everyday home interactions.

### Insistence on the Prohibition against Harming Others

There were three main reasons for hypothesizing heightened maternal insistence on the prohibition against harming others in the present study: (1) The prohibition against harming others is seen as very important by caregivers (vs. pragmatic prohibitions). (2) Harmful acts against others are particularly difficult to prevent (vs. prudential and pragmatic transgressions). (3) Harming others has no direct negative consequences for the transgressor (vs. prudential transgressions). Although some or all of these reasons may also lead mothers from other populations to show heightened insistence on the prohibition against harming others, the hypotheses and study rationale focus on the population under investigation (Rogoff, 2003; see Discussion).

#### The Prohibition against Harming Others Is Important

The general prohibition against harming others is an indispensable part of any social group. This moral norm reflects a fundamental respect for the value of life (Dworkin, 1994). When individuals do harm others, the consequences for transgressors, victims, and those around them are often tragic (Dishion and Patterson, 2006). Indeed, mothers of infants report that they see it as more important to request that their children not harm others than to request that their children not engage in pragmatic, and even prudential, transgressions (Dahl et al., 2014). Mothers also show, and report, more anger in response to moral harm transgressions than in response to prudential and pragmatic transgressions (Dahl and Campos, 2013; Dahl et al., 2014). Some prohibitions against pragmatic transgressions are not even communicated until into the third year (Gralinski and Kopp, 1993; Smetana et al., 2000). Insofar as mothers consider it particularly important to prohibit interpersonal harm in the second year, this provides one reason for hypothesizing high insistence in response to infant moral harm transgressions.

#### Harmful Acts against Others Are Difficult to Prevent

Prevention is one of the most effective ways of keeping children from transgressing. By childproofing the home environment, caregivers can prevent a large number of prudential and pragmatic transgressions (although not all). Baby gates keep children from going down staircases, and locked drawers and cabinets keep children from playing with items that could be dangerous or create a mess (Gärling and Gärling, 1995). Moreover, many prudential and pragmatic transgressions can be anticipated well in advance. If a child is moving toward the pet food container, or an ungated staircase, caregivers will often be able to intervene before the child completes the transgression. In contrast, hitting, biting, and kicking are inherently hard to prevent because these acts are always possible when a child is near another person. Thus, the only way to keep moral harm transgressions from happening in the future may be to try to make the child understand that harming others is not an acceptable behavior. This provides a second reason for expecting heightened insistence on the prohibition against harming others.

#### Harming Others Has No Direct Negative Consequences for the Child

Even minor prudential transgressions are associated with pain or discomfort for the child. Falling off a couch, running into a chair, or touching a hot space heater lead to a direct negative experience that would make the child wary of repeating the transgression. In contrast, hitting others may cause pain to the victim, but not immediately to the transgressor. Thus, a child will be especially reliant on social signals for comprehending the wrongness of harming others. This constitutes a third reason for mothers to be particularly insistent when communicating the prohibition against harming others.

### Forms of Insistence

This study assessed several forms of insistence in mothers’ interventions on moral harm (harming others), prudential (child well-being), and pragmatic (inconvenience) transgressions by 14- to 24-month-olds. These forms of insistence were chosen on the basis of pilot observations, theoretical considerations, and literature review. The subsequent paragraphs describe the maternal behaviors reflecting insistence. A plus sign in parentheses (+) indicates that the behavior represents high insistence (e.g., physical intervention) and a minus sign (-) indicates that the behavior represents low insistence (e.g., distraction).

#### Physical Interventions (+)

*Physical interventions* are highly common in early interactions about transgressions (Zahn-Waxler and Chapman, 1982; Kuczynski et al., 1987; LeCuyer-Maus and Houck, 2002). They are also among the bluntest forms of insistence, leaving little room for negotiation or non-compliance for the physically inferior child. Hence, although physical interventions were expected to be used in all types of interactions about transgressions, they were expected to be especially common after moral harm transgressions (Dahl and Campos, 2013).

#### Direct Commands (+)

The simplest form of verbal intervention is *commands*. While comprehension of commands improves over the second year, Kaler and Kopp (1990) found that infants early in the second year comprehended 30–40 percent of requests for objects. Thus, simple commands could serve as a valuable way of communicating norms to young children.

Commands vary in the level of insistence they convey. Of particular interest in the present study was a distinction between *direct commands* and *indirect commands* (Kuczynski et al., 1987; Crockenberg and Litman, 1990). Direct commands are defined as explicit imperative statements, for instance, “No, don’t hit your brother,” or, “Put the phone down.” Indirect commands are questions or suggestions, for instance, “Do you want to put the phone down?” or “Let’s not hit each other.” Direct commands reflect greater insistence and were hypothesized to be more common in moral harm situations than in prudential and pragmatic situations. In contrast, indirect commands reflect less insistence and were hypothesized to show the opposite pattern.

#### Distraction (-)

*Distraction* is a common way of intervening on infant transgressions (Reid et al., 1994; LeCuyer-Maus and Houck, 2002; Dahl and Campos, 2013). Distractions serve the function of drawing the child’s attention away from the prohibited activity, for instance by attempting to engage the child in play with a toy. Hence, distraction appears to prioritize harmony (preventing the child from getting upset) over communicating to the child that he or she is doing something wrong. For these reasons, distractions were hypothesized to be more common in pragmatic and prudential situations than in moral harm situations (Dahl and Campos, 2013).

#### Softening Intervention (-)

All interactions about transgressions involve some “clash of wills” and hence a chance for infants to get upset (Emde et al., 1987). This forces caregivers to balance the goal of communicating a prohibition with the goal of maintaining a harmonious relationship with the child (Ross, 1996). One way of promoting harmony is to attempt to soften the intervention by acknowledging the child’s desire (e.g., “I know you want to play with my cell phone”), comforting the child (“It’s going to be okay”), or addressing the child with a term of endearment (“Oh, honey”). It was expected that such *softening behaviors* would be more common in prudential and pragmatic situations than in moral harm situations. Mothers will likely be especially prone to seek harmony if the child becomes upset. In pragmatic and prudential situations, but not in moral harm situations, softening responses were thus expected to become more common if the child became visibly distressed.

#### Compromising/Relenting (-)

On average, young children comply with about 60 percent of parental interventions (Kuczynski et al., 1987). Ultimately, it is nearly always possible for caregivers to ensure infant compliance in the end, given caregivers’ superior physical strength. By lifting infants away from the prohibited activity and placing them in a separate space, caregivers can effectively keep infants from transgressing. Hence, in the face of persistent non-compliance, caregivers choose between insisting on compliance (at the risk of making the infant highly distressed) and compromising or relenting. For instance, compromising could mean allowing the child to continue climbing on the couch, or continue playing with a breakable object, while the parent is holding the child’s hand. Compromising or relenting reflects low insistence and was hypothesized to be less common in moral harm situations than in prudential and pragmatic situations.

### Child Responsiveness to Prohibitions

As noted, children do not seem to draw robust categorical distinctions between moral and other violations until around the third birthday (Smetana and Braeges, 1990; Nucci and Weber, 1995; Dahl and Kim, 2014). Still, infants’ responsiveness to prohibitions may yield some indications about whether they perceive differences in caregiver prohibitions against moral and other transgressions (Dahl and Tran, 2016). The present study included two indicators of children’s sensitivity to differences between moral harm, prudential, and pragmatic events: compliance and protests. Young children show high rates of non-compliance with and protests against caregiver requests (Kuczynski et al., 1987). However, if children perceive caregivers as more insistent on the prohibition against harm, they too may begin to “pick their battles,” showing non-compliance and protests in situations in which caregivers previously have shown lower insistence. Thus, it was hypothesized that, in the present study, children would be more likely to comply, and less likely to protest, in moral harm situations than in prudential and pragmatic situations.

### The Present Study

The present study was a longitudinal home observation study of mother–infant conflicts about moral harm, prudential, and pragmatic transgressions during the second year of life. The study differs from past research in several ways. Research investigating mother-infant interactions has typically not distinguished between types of transgressions corresponding to categories of norms constructed by children (Dunn and Munn, 1985; Power and Parke, 1986; Emde et al., 1987; Kuczynski et al., 1987; Dunn, 1988; Kochanska, 2002; LeCuyer-Maus and Houck, 2002). In contrast, studies investigating how moral transgressions elicit interactions different from other transgressions have been typically involved older children, focused on verbal content of interactions (which may be less salient or even incomprehensible to infants), or contrasted only moral and conventional transgressions (Smetana, 1984, 1989; Nucci and Weber, 1995; Tisak et al., 1996; Killen and Smetana, 1999; Turiel, 2008).

The one study that has contrasted mothers’ responses to moral harm, prudential, and pragmatic transgressions in the second year relied on maternal recollections of past interactions (Dahl and Campos, 2013). Aside from being limited by the reliance on maternal report, the study by Dahl and Campos (2013) did not investigate the forms of insistence investigated in the present study. Finally, the study by Dahl and Campos (2013) did not investigate child protests. By using direct, videotaped observations of mother–infant conflicts about these three types of transgressions, and by introducing and studying the concept of caregiver insistence, the present study increases our understanding of the everyday context in which children gradually develop a moral aversion to harming others.

The longitudinal design was chosen to increase the amount of data obtained from each family and to take advantage of the increased statistical power of within-subjects designs. Hence, the target sample size (*N* = 26) and the frequency of visits (three, one every 5 months) of this study were not chosen with the aim of investigating individual differences or individual patterns of change. While additional studies with larger sample sizes and more data from each participant will be needed to investigate individual differences (see Discussion), the goal of this initial longitudinal study was to investigate overarching trends in mother–infant interactions about moral harm, prudential, and pragmatic transgressions.

## Materials and Methods

This study was carried out in the accordance with the recommendations of the University of California, Berkeley Committee for the Protection of Human Subjects (UCB CPHS). Parents of participating infants provided written consent in accordance with the Declaration of Helsinki. The research was approved by UCB CPHS (protocol number 2010-04-1348).

### Participants

Twenty-six families participated in a 2.5-h home visit when the target child (11 female, 15 male) was 14 months of age (*M*_age_ = 14.5 months, *SD*_age_ = 0.63 months). Most families participated in a second visit when the child was 19 months (*N* = 24, *M*_age_ = 19.5 months, *SD*_age_ = 0.59 months) and again when the child was 24 months (*N* = 22, *M*_age_ = 24.5 months, *SD*_age_ = 0.51 months). Two families were too busy to participate in subsequent visits, one family moved to another state, and one family stopped speaking English at home. All target children had at least one older sibling less than 8 years of age. Families were recruited from the participant database at the University of California, Berkeley. The database contained contact information of families living in a metropolitan area in the Western US who had previously expressed interest in participating in research. Sixty-three percent of parents were non-Hispanic Caucasian, 19 percent were Asian American, and 19 percent were of African–American, Hispanic, other, or mixed ethnicity. Seventy-seven percent of caregivers had a college degree and 29 percent had a graduate degree.

### Materials and Procedure

At all visits, the target child, the mother, and one older sibling less than 8 years of age were present. The requirement of having one older sibling present was included to make the family context more comparable for the different families. Visits were scheduled to include a mealtime, also to keep the observation context more comparable across visits.

Mothers were told to do whatever they would have been doing if the observer had not been present, as the purpose of the study was to investigate children’s everyday experiences. During the visit, the observer videotaped the child’s behavior while remaining at a distance and avoiding interactions with family members. The observer logged every instance of the mother intervening on the target child’s behavior, either by trying to stop the target child from doing something or by negatively evaluating the child’s action. The logging was done electronically using an *iPod Touch* (Apple, Inc.). Three observers collected data for the study. Observers were trained using pilot video recordings until they logged 80 percent of the events logged by the main observer. The purpose of the logging procedure was to combine the advantages of event sampling (for the purposes of initial data reduction) and coding of videotaped interactions (allowing for detailed coding of naturalistic interactions).

At the first visit, mothers were given a demographics questionnaire. At all three visits, mothers were also given a behavior rating questionnaire. On the behavior rating questionnaire, mothers indicated how important it was for them to request that their child refrain from certain behaviors on a scale from 1 (*unimportant*) to 4 (*very important*). The list of behaviors was taken from Gralinski and Kopp (1993). Only ratings pertaining moral harm (not being rough with other children, not biting mom or dad), prudential (not climbing on furniture, not going into the street, not touching things that are dangerous), and pragmatic (not coloring on walls, not tearing up books, not interrupting, not playing with food, not spilling drinks) transgressions were included. Ratings for behaviors in each category were averaged to create one moral, one prudential, and one pragmatic score for each mother for each visit.

After the visit, the observer watched the logged situations in the video recordings to identify situations in which there was any doubt about why the mother intervened (for instance, when the mother stopped the child from opening a cabinet but the observer did not know what the cabinet contained). The observer then conducted a phone interview with the mother about these situations (typically 30–40 percent of situations), in which the interviewer asked the mother what her concern was in intervening upon the child’s behavior. Summaries of the mothers’ responses for each situation were used for classifying the child’s transgression (see Coding and Data Analysis).

### Coding and Data Analysis

The video recordings obtained during the home visits were coded by research assistants blind to the study hypotheses. Coders only coded situations that had been logged during the observation. Video recordings from 20 percent of visits were coded by two independent coders to assess inter-rater agreement.

Three sets of codes were applied to the interactions about transgressions (situations in which the mother negatively evaluated or attempted to stop the child’s behavior). One set of codes applied to the level of the *situation* as a whole, a second set of codes applied to the *mother’s individual actions* within the situation, and a third set applied to the *child’s individual actions* within the situation.

#### Situation Codes

Child transgressions were coded as either *moral harm* (causing interpersonal harm, for instance hitting, biting, or kicking someone else), *prudential* (doing something dangerous, for instance climbing on furniture or putting toys in mouth), *pragmatic* (causing inconvenience but no potential or actual harm, for instance creating disorder, spilling food, or playing with a breakable object), or *other*. Situations classified as “other,” involving for instance conventional violations (2%) and property conflicts (3%), were rare (total: 6% of situations) and were not included in the analyses. If a mother had been interviewed about the situation, the coder classified the situation based on the mothers’ explanation. Situations involving both pragmatic and moral *or* prudential concerns were classified as moral or prudential respectively. Only 87 such mixed events were recorded (4.7% of all events). Situations involving both moral and prudential were rare and were excluded from analysis (*N* = 2). Agreement for situation classification: κ_Cohen_ = 0.90.

Coders also assessed how far the child had gotten in the transgressive activity at the time of the first intervention (“degree of completion:” *started* or *completed*, κ_Cohen_ = 0.79). For instance, if a child was reaching for a prohibited object on the table at the time when the mother intervened, the degree of completion would have been coded as *started*. In contrast, if the child had already pushed the object off the table and onto the floor, the degree of completion would have been coded as *completed*. The purpose of this coding was to check the assumption that intervention after completion of at least one transgressive act was more common in moral harm situations than in prudential and pragmatic situations.

Finally, coders assessed what the outcome of the situation was (*child complies*, *compromise*, or *mother relents*, κ_Cohen_ = 0.79).

#### Mother and Infant Codes

Codes for mothers’ and infants’ actions were derived from previous studies of early family interactions (Kuczynski et al., 1987; Smetana, 1989; Nucci and Weber, 1995; LeCuyer-Maus and Houck, 2002; Dahl and Campos, 2013) as well as from pilot observations. The codes were taken as indices of maternal insistence (see Introduction), except for verbal explanations, which were included to check the assumption that mothers’ viewed moral harm, prudential, and pragmatic transgressions as qualitatively different. Since these codes were applied to individual actions, multiple codes could be used for a single interactant in a single situation (e.g., a mother could use both physical intervention and verbal explanation in a single situation).

For every maternal action within a transgressive situation the coder noted presence of the following: *physical intervention*, *verbal command* (either *direct command* or *indirect command*), *distraction, softening*, and *verbal explanation* (see **Table 1** for definitions). Explanations were further classified using the following coding categories derived from pilot observation and past research: *Disorder, evaluation, feelings/desires of others, harm to child, harm to others, property ownership, rule, sanction, and other* (Nucci and Turiel, 1978; Smetana, 1989; Nucci and Weber, 1995; Dahl and Campos, 2013). Mean agreement for maternal interventions: κ_Cohen_ = 0.76 (range: 0.68–0.91). The infants’ behavior was coded for the following: *compliance* with the initial maternal intervention (i.e., stopped the behavior after first intervention), *protest* during the situation (e.g., by saying “no” or “a little longer”), or vocal or facial signs of *negative emotion* during the situation. Mean agreement for child codes: κ = 0.82 (range: 0.65–0.98).

**Table 1:** List of maternal intervention codes.

Code	Definition
Physical intervention	Use of force to stop or prevent child from transgressing.
Direct command	Explicit command telling the child to alter behavior, including second-person imperative verb form (“Don’t hit your brother,” “Put the scissors down”).
Indirect command	Suggestive or mild command telling the child to alter behavior, including first-person plural imperative (“Let’s not play with that”) or question (“Do you want to give me the scissors?”).
Softening	Acknowledgment of infant’s desire, attempt to comfort the child, proposal of compromise, term of endearment (e.g., “honey”).
Distraction	Attempt to draw child’s attention to something other than the prohibited behavior.
Verbal explanation	Verbal statement indicating why a given behavior was wrong.
*Verbal explanations were further classified into one of the following categories:*	
Disorder	Potential or actual disorder or property damage, including mess or spilling (e.g., “Look at this mess”).
Evaluation	Evaluative statement about act (e.g., “That’s not very nice”)
Feelings/desires	Potential or actual feelings or desires of another person (excluding pain or harm, e.g., “That’s annoying me”).
Harm to child	Potential or actual harm to the child (e.g., “You’ll hurt yourself”).
Harm to others	Potential or actual harm to another person (e.g., “That’ll hurt your sister”).
Property ownership	Ownership of object (e.g., “That’s mommy’s cup”).
Rule	Wrongness of act described on a general level (“We don’t run in the stairs”).
Sanction	Potential or actual sanction against the child (“One more and you’ll get a time-out”).
Other reason	Any reason not fitting into the above categories (“We’re going to go to Grandpa’s house now”).

#### Seriousness of Physical Harm

To investigate whether heightened insistence on moral harm prohibitions compared to prudential prohibitions could be explained by the seriousness of physical harm, coders assessed the seriousness of potential or actual harm associated with each prudential and moral transgression. Codes were applied based on the harm that could have occurred if the child continued transgressing rather than the harm that actually occurred, since children were often stopped from completing the transgression (e.g., stopped before being hit by a car). Each of the types of harm was later assigned a numerical value representing the relative seriousness of the immediate consequences. Immediate (rather than long-term) consequences were emphasized because it was expected that mothers would be more insistent if consequences were immediate (e.g., falling down from a high-chair) than when consequences required prolonged exposure to the hazard (choking or, even more long-term, sunburn).

Based on review of the types of transgressions encountered in the visits, the following codes were used: (The numerical seriousness values are listed in parentheses.) *Hit by car* (5), *falling down from elevated location* (e.g., kitchen counter) (4), *choking/suffocation* (3), *burn or cut injuries* (3), *being hit, bitten, kicked*, *or stepped on* (2), *falling down on flat surface* (2), *detectable harm only with repeated exposure* (e.g., sunshine, 1). In principle, both moral and prudential transgressions could be coded at any level of severity (1–5). Agreement for numerical ratings: Pearson’s *r* = 0.87.

#### Data Analytic Strategy

Data were analyzed using Generalized Linear Mixed Models (GLMMs) with Poisson, Gaussian, or binomial error distributions and logarithmic, identity, or logistic link functions respectively (Hox, 2010). Models were fitted in R version 3.2.4 using the lme4 package. Initial analyses revealed no significant effects of child gender, which was therefore not included as a predictor in the final models. Random effects of visit and family id were used to account for the nested structure of the data. The specific models used for each dependent variable are described in Section “Results.”

For each form of insistence, the percentage of mothers showing the predicted pattern (greater average insistence in moral harm situations) is reported, controlling for visit number, to indicate whether most families showed the overall pattern of situational differences.

## Results

A total of 1861 situations involving moral harm, prudential, or pragmatic transgressions were coded in the 72 visits, yielding an average frequency of 10.3 situations per hour. Among these situations, 120 (6.4%) were coded as moral harm, 699 (37.6%) as prudential, and 1042 (56.0%) as pragmatic. The targets of children’s moral transgressions were parents (40%), siblings (43%), and pets (17%). Twenty-one infants had at least one moral situation (19/22 of families with three visits) and all 26 infants had at least one prudential situation and one pragmatic situation.

### Mothers’ Verbal Explanations

Mothers’ provided explanations in 41% of situations. There was no significant effect of situation type on whether mothers provided at least one explanation in a given situation, *D*(2) = 1.39, *p* = 0.50. The use of explanations had a near-significant, positive relation to infant age, *D*(1) = 3.82, *p* = 0.051. However, the main hypotheses about explanations pertained to the association between the content of explanations and the nature of the child’s transgression.

Mothers’ verbal explanations in response to transgressions supported the hypothesis that mothers draw qualitative distinctions between moral harm, prudential, and pragmatic violations (Smetana et al., 2000; Dahl and Campos, 2013). The type of justification provided by mothers dependent significantly on situation type, binomial GLMM: *D*(12) = 460.80, *p* < 0.001. (Only data from situations in which the mother provided at least one justification were included and models included child age as predictor.) Consistent with past work, references to harm to others were most common in moral situations (63% of moral situations), references to harm to the child were most common in prudential situations (44%) and references to disorder or property destruction were most common in pragmatic situations (25%). Reasons classified as “other” were more common in pragmatic situations (45%) than in moral situations (20%) with prudential falling in between. This category included justification statements not falling into any of the other justification categories, such as “Don’t get your toys out now because we’re going to Grandpa’s house.” Anecdotally, it appeared that many of the explanations classified as “other” expressed mothers’ pragmatic considerations (i.e., about inconvenience of child’s behavior for ongoing or planned activities). **Table 2** shows how the justification categories varied as a function of situation type.

**Table 2:** Content of explanation as a function of situation type.

	Situation type		
	Moral harm	Prudential	Pragmatic
Disorder	0.00_a_	0.04_a_	0.25_b_***
Evaluation	0.13	0.21	0.16
Feelings/desires	0.00	0.05	0.07
Harm to child	0.00_a_	0.44_b_	0.01_a_***
Harm to others	0.63_a_	0.00_b_	0.01_b_***
Property ownership	0.02	0.01	0.05
Rule	0.11	0.17	0.11
Sanction	0.09	0.06	0.05
Other reason	0.20_a_	0.29_a,b_	0.45_b_***

The table shows number of situations in which the mother used a particular explanation type divided by the number of situations in which a mother used any explanation, calculated separately for each domain. P-values are adjusted for multiple tests using Holm’s (1979) procedure. Cells with different subscripts differ significantly (adjusted p < 0.05). *** Situation effect is significant (adjusted p <.001).

### Degree of Completion

Whether the child had completed at least one transgressive act before the first maternal intervention (a dichotomous variable) was analyzed using binomial GLMMs with random intercepts for visit and family and fixed effects of situation type and child age. Hypotheses for main effects of situation and age were tested using likelihood ratio tests (for difference in model deviance between full model and model with the relevant coefficients constrained to 0) and Wald tests were used to compare each situation type (Hox, 2010).

As expected, there was a significant effect of situation type on degree of completion before first maternal intervention, *D*(2) = 41.90, *p* < 0.001. Interventions after completion of at least one transgressive act were more common in moral situations (33%) than in prudential (7%) and pragmatic (10%) situations, Wald tests: *p*s < 0.001. Intervention after completion was also more common when infants were older, *D*(1) = 6.00, *p* = 0.01 (Visit 1: 8%, Visit 2: 10%, Visit 3: 14%).

### Ratings of Importance of Requests

Importance ratings were analyzed using Gaussian GLMMs with random intercepts for visit and family and fixed effects of situation type and child age. Hypotheses for main effects of situation and age were tested using likelihood ratio tests and Wald tests were used to compare each situation type.

Mothers said it was more important to request that their infants not engage in moral harm transgressions (*M* = 3.76, *SD* = 0.38) than to request that their infants not engage in prudential (*M* = 3.59, *SD* = 0.34) or pragmatic transgressions (*M* = 2.82, *SD* = 0.53), Gaussian GLMM: *D*(2) = 140.95, *p* < 0.001. Wald tests revealed that ratings of all three situation types differed significantly from each other, *p*s < 0.01. Overall, mothers’ importance ratings increased with child age, *D*(1) = 14.01, *p* < 0.001 (Visit 1: 3.27, Visit 2: 3.36, Visit 3: 3.53).

### Maternal Insistence

**Table 3** shows a summary of the findings on the five forms of insistence.

**Table 3:** Overview of findings on forms of insistence.

	Situation Type			Situation effect
	Moral	Prudential	Pragmatic	
Physical interventions	0.31	0.31	0.21	–
*Prop. of interventions*	*0.18*_a_	*0.15*_b_	*0.11*_c_	***
Direct commands	0.78	0.75	0.70	–
*Prop. of commands*	*0.90*_a_	*0.76*_b_	*0.73*_c_	***
Distraction	0.04_a_	0.11_b_	11_b_	^∗^
Softening				
*Child distress*	0.08_a_	0.41_b_	0.51_b_	**
*No distress*	0.14_a_	0.27_b_	0.22_a,b_	*
Compromise/relenting	0.03_a_	0.07_b_	0.10_b_	***

*The table shows an overview of the findings for the five forms of insistence. The columns for situation type indicate the proportion of situations that included a given type of maternal intervention. Since data for physical interventions and direct commands were analyzed by individual interventions and commands respectively (rather than by situations, see Results), the proportions corresponding to the analyses reported in the text are provided in italics. Cells with different subscripts differ significantly at p < 0.05 (Wald tests for regression coefficients). The rightmost columns indicate significance levels for overall effects of situation obtained from likelihood ratio tests. ^∗^p < 0.05, ^∗∗^p < 0.01, ^∗∗∗^p < 0.001.*

The high prevalence of *physical interventions* made it possible to analyze these data at the level of individual interventions rather than at the level of situations. Whether a mother used a physical intervention was predicted using binomial GLMMs with random intercepts for visit and family and fixed effects of situation type, intervention number (within each situation), and child age. Hypotheses for main effects of situation and age were tested using likelihood ratio tests and Wald tests were used to compare each situation type. As the number of maternal actions varied between situations (range: 1–27), only the five first actions were included in the analyses, which included 75 percent of interventions.

As predicted, there was a significant effect of situation type on the use of physical interventions, binomial GLMM: *D*(2) = 20.07, *p* < 0.001. Wald tests revealed that the use of physical interventions was significantly more common in moral harm situations (18% of interventions were physical) than in prudential (15%) and pragmatic situations (11%), *p*s < 0.05. Physical interventions were also significantly more common in prudential than in pragmatic situations. At the level of individuals, 43% of mothers were more likely to use physical interventions in moral situations than in prudential and pragmatic situations. The presence of physical interventions was also more common later in the situation (i.e., positively related to intervention number), *D*(1) = 34.78, *p* < 0.001, and negatively associated with child age, *D*(1) = 8.11, *p* = 0.004.

The main question regarding *commands* was whether the type of command (direct vs. indirect) depended on situation type. To test this hypothesis, binomial GLMMs were fitted to predict whether the command used was a direct or an indirect command (only interventions involving maternal commands were included). The models also included random intercepts for visit and family and fixed effects of situation type, intervention number, and child age. Hypotheses for main effects of situation and age were tested using likelihood ratio tests and Wald tests were used to compare each situation type. As in the analyses of physical interventions, only the first five interventions from each situation were included in the analyses.

There was a significant effect of situation type, *D*(2) = 39.18, *p* < 0.001, in predicting the type of command used. Direct (as opposed to indirect commands) were significantly more common in moral harm situations (90.2% of commands) than in prudential (75.8%) and pragmatic (72.7%) situations, Wald tests: *p*s < 0.001. Direct commands were in turn more common in prudential than in pragmatic situations, χ^2^(1) = 5.8, *p* = 0.016. Seventy-six percent of mothers were more likely to use direct commands in moral situations than in prudential and pragmatic situations, while 90% of mothers were more likely to use indirect commands in prudential and pragmatic situations than in moral situations (after correcting for intervention number and visit number). Direct commands were also more common earlier in the interaction, *D*(1) = 12.97, *p* < 0.001, but were not significantly associated with child age, *D*(1) = 0.46, *p* = 0.30.

Due to the lower frequency of the remaining forms of insistence (distraction, softening, and relenting/compromising, occurring in less than 10% of situations for at least one situation type), it was not possible to fit models for these variables that included intervention number. The models analyzing these insistence forms therefore analyzed whether these interactions were present or absent from a situation using binomial GLMMs with random intercepts for visit and family and fixed effects of situation type and child age.

Attempts to *distract* the child were significantly less common in moral harm situations than in prudential and pragmatic situations, binomial GLMM: *D*(2) = 6.17, *p* = 0.046. Wald tests showed that moral situations (4.2%) differed significantly from prudential (10.9%) and pragmatic situations (11.0%), *p*s < 0.05, whereas the two latter did not differ from each other, χ^2^(1) = 0.26, *p* = 0.61. At the level of individuals, 90% (19/21) of mothers used distraction more often in prudential and pragmatic situations than in moral situations. The use of distractions was also negatively related to child age, *D*(1) = 4.91, *p* = 0.027 (Visit 1: 16.7%, Visit 2: 7.9%, Visit 3: 6.7%).

Also in line with hypotheses, there was a significant interaction between situation type and infant negative emotion for *softening interventions*, *D*(2) = 6.22, *p* = 0.045. In prudential and pragmatic situations, softening was significantly more common if the child showed negative emotion (Prudential: *D*[1] = 5.13, *p* = 0.024, Pragmatic: D[1] = 39.41, *p* < 0.001). In contrast, there was no relation between infant negative emotion and maternal softening in moral harm situations, *D*(1) = 0.56, *p* = 0.45. The effect of situation type was significant for situations with negative emotion, *D*(2) = 10.49, *p* = 0.005, and without negative emotion, *D*(2) = 7.19, *p* = 0.027. In both cases, moral harm situations elicited the least softening interventions. Ninety percent of mothers used softening more often in prudential or pragmatic situations than in moral situations.

Finally, mothers’ tendency to *compromise or relent* was also significantly related to situation type, binomial GLMM: *D*(2) = 13.82, *p* < 0.001. Compromising or relenting was less common in moral harm situations (2.5%) and prudential situations (7.0%) than in pragmatic situations (10.3%), Wald tests: *p*s < 0.02, whereas moral harm and prudential situations did not differ significantly, *χ2*(1) = 2.40, *p* = 0.12. Ninety percent of mothers were more likely to relent or compromise in prudential or pragmatic situations than in moral harm situations. There was no significant effect of child age, *D*(1) = 0.27, *p* = 0.60.

### Child Reactions to Interventions

#### Child Protests

Whether a child protested in a given situation was analyzed using binomial GLMMs with random intercepts for visit and family and fixed effects of situation type and child age. Hypotheses for main effects of situation and age were tested using likelihood ratio tests and Wald tests were used to compare each situation type.

There was a significant effect of situation on the presence of child verbal protest, binomial GLMM: *D*(2) = 6.72, *p* = 0.035. Infants protested significantly more in pragmatic situations (7.2% of situations) than in moral harm situations (3.3%), Wald test: χ^2^(1) = 4.5, *p* = 0.034. The probability of protest in prudential situations (5.2%) did not differ significantly from that in moral situations, χ^2^(1) = 2.2, *p* = 0.13, or pragmatic situations, χ^2^(1) = 2.0, *p* = 0.16. There was also an effect of child age, *D*(1) = 9.16, *p* = 0.002 (Visit 1: 1.0%, Visit 2: 6.2%, Visit 3: 12.1%). The interaction between child age and situation type was not significant, *D*(2) = 0.04, *p* = 0.98.

#### Immediate Child Compliance

Whether a child initially complied in a situation was analyzed using binomial GLMMs with random intercepts for visit and family and fixed effects of situation type and child age. Hypotheses for main effects of situation and age were tested using likelihood ratio tests and Wald tests were used to compare each situation type. These analyses included all situations, including those in which children had already transgressed once, because it was nearly always possible for children to transgress a second time (e.g., by hitting again) and thus failing to comply with the maternal prohibition.

There was a significant effect of situation type on immediate compliance, binomial GLMM: *D*(2) = 8.54, *p* = 0.010. Immediate compliance was higher in moral harm situations (59%) than in prudential (42%) and pragmatic (46%) situations, Wald tests: *p*s < 0.05. Prudential and pragmatic situations did not differ significantly, Wald test: χ^2^(1) = 2.8, *p* = 0.10. There was no significant effect of child age on immediate compliance, *D*(1) = 1.00, *p* = 0.32. The differences in compliance were not simply due to differences in maternal use of physical interventions, as immediate compliance was highest in moral situations also when only analyzing data from situations with no physical interventions, *D*(2) = 8.54, *p* = 0.014.

### Seriousness of Harm

One possible explanation for findings of greater insistence in moral situations is that moral situations involved more serious potential or actual harm than prudential situations. (By definition, pragmatic situations did not involve harm.) To test this possibility, a linear mixed model was fitted to the data from prudential and moral harm situations with Gaussian error distribution and identity link function predicting severity rating as a function of child age and situation type. There was a significant effect of situation type, *D*(1) = 508.03, *p* < 0.001, as prudential situations involved significantly greater potential or actual harm (*M* = 2.94) than moral harm situations (*M* = 2.05). There was also a significant effect of child age, *D*(1) = 4.45, *p* = 0.034, reflecting a positive relation between child age and potential or actual harm. On an individual level, 95% of families had higher mean severity of prudential than of moral situations, binomial test: *p* < 0.001. In short, any heightened insistence in moral situations compared to prudential situations could not be explained by greater overall severity of harm in moral situations.

## Discussion

The present study investigated mothers’ insistence when intervening on infants’ moral, prudential and pragmatic transgressions. The main hypothesis was that mothers would show greater insistence on moral prohibitions (against harming others) than on prudential prohibitions (against doing something that could affect the child’s welfare) and pragmatic prohibitions (against creating inconvenience). The rationale for this hypothesis was supported by several findings. First, mothers reported that moral requests were more important than prudential and pragmatic requests. Second, mothers were more likely to intervene prior to completion of a transgressive act in the prudential and pragmatic situations than in the moral situations. As noted in the Introduction, since moral transgressions are less preventable, the primary way to stop moral transgressions from happening may be to convey to the child that the behavior is not acceptable.

As hypothesized, mothers showed greater insistence in moral harm situations than in prudential and pragmatic situations across several forms of insistence. In response to moral transgressions, mothers showed greater use of physical interventions (e.g., holding the child back), greater use of direct commands (“No, don’t hit your brother”), decreased use of distraction (e.g., drawing child’s attention to something else), and decreased use of softening interventions (e.g., acknowledging child’s desire to transgress). In prudential and pragmatic situations, but not in moral harm situations, mothers were more likely to use softening interventions if the child became upset. Finally, mothers were four times more likely to compromise or relent in pragmatic situations than in moral harm situations, with prudential situations falling between the two.

The greater insistence in moral situations compared to prudential situations was not attributable to greater severity of harm in moral situations. On the contrary, the actual or potential harm was, on average, more severe in prudential situations than in moral situations. This finding is particularly striking since Western caregivers prevent many severe prudential transgressions from happening through babyproofing their homes (Gärling and Gärling, 1995).

Mothers’ overall heightened average insistence on the prohibition against harming others likely contributes to children’s growing ability to distinguish moral, prudential, and pragmatic transgressions (Smetana and Braeges, 1990; Tisak, 1993; Smetana et al., 2012, 2014; Dahl and Kim, 2014). Heightened insistence in moral situations may communicate to infants that mothers viewed harming others as a particularly serious transgressions. Heightened insistence may also draw infants’ attentions to the categorically distinct features of moral harm transgressions. That is, insofar as more insistent reactions are more likely to call infants’ attention to their violation, mothers’ insistence may help children realize that the use of force against others causes pain or discomfort. Finally, some forms of insistence may lead children to view prohibitions as varying in how negotiable they are. For instance, if children experience that parents are comforting them when they cannot continue with a pragmatic transgression (*softening*) or are allowing children to continue transgressing after an initial prohibition (*relenting*), this may lead children to view pragmatic rules as subject to negotiation and alterable, unlike moral rules (Nucci and Weber, 1995; Dahl and Kim, 2014). (I thank an anonymous reviewer for this suggestion.)

The data on child compliance and protests were consistent with the claim that young children are sensitive to the differences between moral harm, prudential, and pragmatic prohibitions. Infants showed greater immediate compliance with, and lower tendency to protest against, mothers’ prohibitions against harming others, compared to their reactions in prudential and pragmatic situations. While limit-testing is common during the early years (Dunn and Munn, 1985; Kuczynski et al., 1987), children’s perceptions of transgressions and expectations about caregiver behavior may guide children’s decisions about when to protest and comply (Dahl and Tran, 2016).

Mother’s heightened insistence on prohibitions against moral harm transgressions may also have contributed to why infants engaged in more prudential and pragmatic transgressions than moral harm transgressions. However, it is difficult to compare these frequencies directly since the number of opportunities to engage in these transgressions may also differ. In a typical home, there are far more objects, with which infants could create a mess, than there are people and pets, which infants could harm. Moreover, people afford many acts aside from harming (a parent can be hugged, played with, or watched), whereas many objects may afford few non-transgressive behaviors to infants (food can be eaten, spilled, or taken away) (Gibson, 1979).

Maternal insistence is only one of several features of social experience that may help children develop distinct concepts about harm transgressions. Consistent with past research, mothers in the present study provided different verbal explanations to different transgressions, for instance referring to others’ wellbeing following moral harm transgressions (e.g., Smetana, 1984, 1989; Nucci and Weber, 1995). Moreover, moral harm transgressions often involve expressions of distress from a victim and children may be involved in such events not only as transgressors but also as victims of others’ transgressions (Turiel, 1983; Zahn-Waxler et al., 1992). Situational features may vary in how informative they are to children at various ages. For instance, infants may not fully understand the differences between verbal explanations uttered in response to moral, prudential, and pragmatic transgressions (Fenson et al., 1994; Dahl and Campos, 2013). Ultimately, it seems likely that children must draw on multiple sources of converging information (e.g., caregiver insistence, verbal explanations, own experiences of pain, observations of others’ distress) in order to construct categorically distinct moral, prudential, and pragmatic concepts.

Two types of additional research would be particularly important to advance our knowledge about how children construct moral, prudential, and pragmatic concepts during the second and third years of life. First, larger naturalistic studies are needed do document different developmental trajectories and predict individual differences. While the present study focused on overarching differences in children’s experiences following different transgressions, and lacked the sample size and the number of data points to meaningfully investigate individual differences and trajectories, the theoretical framework that motivated this research yields testable predictions about individual differences (Smetana et al., 2014). For instance, with a larger number of families and more frequent visits, it would be possible to investigate whether children whose parents respond more differentially to moral and other transgressions show earlier signs of differentiating between moral and other prohibitions, as indicated by differential levels of transgressions, compliance, or protest rates. Indeed, past research has found stable individual differences in rates of aggressive behaviors starting in the toddler years (Cummings et al., 1989; Keenan and Shaw, 1994). There may also be individual differences in the ability to distinguish between moral and other types of transgressions. Most children eventually draw such distinctions, but the development of domain distinctions may follow different trajectories (Smetana and Braeges, 1990; Jagers et al., 1996; Smetana et al., 2012). By following children into the third and fourth years, it would be possible to predict individual differences in the development of children’s ability to verbalize distinctions between moral and other norms, as shown by Smetana et al. (2012).

Secondly, additional experimental research can elucidate how young children construct moral, prudential, and pragmatic conceptions from everyday experiences. [For discussions of the complementary roles of naturalistic and experimental research, see Bandura and Walters (1963); Willems (1967), Gibson (1979).] By manipulating the situational features of situations, e.g., the mothers’ reaction to an event, it is possible to test how children make use of such information (Dahl and Tran, 2016). For instance, one experiment manipulated whether preschoolers saw a novel action either cause pain to a puppet and elicit a prohibition from an adult or cause a box to make a sound and elicit a prohibition from an adult (Srinivasan et al., 2015). Children who saw the act cause pain and elicit a prohibition viewed the act as a moral transgression, whereas children who saw the act make a sound and elicit a prohibition viewed the act as a conventional transgression (or no transgression at all).

Past research has demonstrated cultural and contextual variability in when and how parents from different communities communicate prohibitions to their families (Dunn and Brown, 1991; Harkness and Super, 2002; Rogoff, 2003). Briggs (1974) noted that the Inuit families she observed rarely explicitly corrected misbehaviors when children were very young. [For similar reports from other communities of adults “indulging” young children, see Hewlett (1992) and Chapin (2010).] Within a given community, the presence of siblings may affect the rate of conflicts, especially moral ones (Tremblay et al., 1999). It is also possible that observations in other settings, such as playgrounds, would change the distribution of events, for instance by increasing the number of moral transgressions or by allowing for other types of transgressions within each category (e.g., picking up trash from the ground or hitting unfamiliar peers). While a single study cannot study all the relevant natural contexts in which children engage in transgressions, a concerted effort involving multiple studies and investigators will ultimately provide a more complete picture of the variabilities (and similarities) in the many everyday contexts of early socio-moral development than we currently have.

Despite such contextual variability, some basic features likely characterize most caregiver-child relationships. First, as mentioned, most infants occasionally use force against others (Dunn and Munn, 1985; Tremblay et al., 1999; Dahl and Campos, 2013). Second, the three reasons for expecting heightened average insistence on the prohibition against harming others apply, albeit to varying degrees, across contexts: (1) The prohibition against harming others is central to most parents of young children (unlike pragmatic transgressions), (2) infants’ moral transgressions are harder to prevent than prudential and pragmatic transgressions, and (3) moral transgressions lack direct negative consequences for the transgressor (unlike prudential transgressions) (LeVine, 1988; Turiel, 2005).

This naturalistic study suggests that mothers generally show greater insistence on the moral prohibition against harming others than on prudential and pragmatic prohibitions. Additional research is needed to investigate individual and cultural variability in caregivers’ differentiated responses to transgressions, as well as how children make use of caregivers’ responses to construct the distinct conceptions of moral, prudential, and pragmatic norms evident in most children by preschool-age (Smetana and Braeges, 1990; Tisak, 1993; Nucci and Weber, 1995; Dahl and Kim, 2014).

## Author Contributions

AD designed the study, supervised data collection and coding, analyzed the data, and wrote the manuscript.

## Conflict of Interest Statement

The author declares that the research was conducted in the absence of any commercial or financial relationships that could be construed as a potential conflict of interest.

## Footnotes

1 The present study focused on moral harm events rather than all moral events, which by most definitions also include issues of property rights and fairness (Turiel, 1983, 2015; Smetana, 2013). There were two reasons for this. First, past research by Ross (1996) has indicated that parents do not insist on particular principles of property (possession vs. ownership) during early family interactions, prioritizing harmony over the enforcement of property rights. Second, pilot observations suggested that interactions about other moral transgressions (e.g., violations of fairness) were so rare that the present study would not have yielded enough data to analyze responses to such transgressions.

## References

[B1] Bandura A. Walters R. H. (1963). *Social Learning and Personality Development.* New York, NY: Holt, Rinehart, & Winston.

[B2] Biringen Z. Emde R. N. Campos J. J. Appelbaum M. I. (1995). Affective reorganization in the infant, the mother, and the dyad: the role of upright locomotion and its timing. *Child Dev.* 66 499–514. 10.2307/1131593 7750380

[B3] Bloom P. (2013). *Just Babies: The Origins of Good and Evil.* New York, NY: Crown.

[B4] Briggs J. L. (1974). *Never in Anger: Portrait of an Eskimo family.* Cambridge, MA: Harvard University Press.

[B5] Chapin B. L. (2010). “We have to give”: sinhala mothers’ responses to children’s expression of desire. *Ethos* 38 354–368. 10.1111/j.1548-1352.2010.01155.x

[B6] Crockenberg S. Litman C. (1990). Autonomy as competence in 2-year-olds: maternal correlates of child defiance, compliance, and self-assertion. *Dev. Psychol.* 26 961–971. 10.1037/0012-1649.26.6.961

[B7] Cummings E. M. Iannotti R. J. Zahn-Waxler C. (1989). Aggression between peers in early childhood: individual continuity and developmental change. *Child Dev.* 60 887–895. 10.2307/1131030 2758884

[B8] Dahl A. Campos J. J. (2013). Domain differences in early social interactions. *Child Dev.* 84 817–825. 10.1111/cdev.12002 23106238

[B9] Dahl A. Campos J. J. Witherington D. C. (2011). Emotional action and communication in early moral development. *Emot. Rev.* 3 147–157. 10.1177/1754073910387948

[B10] Dahl A. Kim L. (2014). Why is it bad to make a mess? Preschoolers’ conceptions of pragmatic norms. *Cogn. Dev.* 32 12–22. 10.1016/j.cogdev.2014.05.004 PMC5283392 28154468

[B11] Dahl A. Sherlock B. R. Campos J. J. Theunissen F. E. (2014). Mothers’ tone of voice depends on the nature of infants’ transgressions. *Emotion* 14 651–665. 10.1037/a0036608 24866527

[B12] Dahl A. Tran A. Q. (2016). Vocal tones influence young children’s responses to prohibitions. *J. Exp. Child Psychol.* 152 71–91. 10.1016/j.jecp.2016.07.009 27518810 PMC5053893

[B13] Dishion T. J. Patterson G. R. (2006). “The development and ecology of antisocial behavior in children and adolescents,” in *Developmental Psychopathology: Risk, disorder, and adaptation* Vol. 3 2nd Edn, eds Cicchetti D. Cohen D. J. (Hoboken, NJ: John Wiley & Sons), 503–541.

[B14] Dunn J. (1988). *The Beginnings of Social Understanding.* Oxford: Blackwell.

[B15] Dunn J. Brown J. (1991). “Becoming American or English? Talking about the social world in England and the United States,” in *Cultural Approaches to Parenting*, ed.Bornstein M. H. (Hillsdale, NJ: Lawrence Erlbaum Associates, Inc), 155–172.

[B16] Dunn J. Munn P. (1985). Becoming a family member: family conflict and the development of social understanding in the second year. *Child Dev.* 56 480–492. 10.2307/1129735

[B17] Dworkin R. (1994). *Life’s Dominion: An Argument about Abortion, Euthanasia, and Individual Freedom.* New York, NY: Vintage Books.

[B18] Emde R. N. Johnson W. F. Easterbrooks M. A. (1987). “The do’s and don’ts of early moral development: psychoanalytic tradition and current research,” in *The Emergence of Morality in Young Children*, eds Kagan J. Lamb S. (Chicago, IL: University of Chicago Press), 245–276.

[B19] Fenson L. Dale P. S. Reznick J. S. Bates E. Thal D. J. Pethick S. J. (1994). Variability in early communicative development. *Monogr. Soc. Res. Child Dev.* 59 1–173. 10.2307/1166093 discussion 174–185, 7845413

[B20] Gärling A. Gärling T. (1995). Mothers’ anticipation and prevention of unintentional injury to young children in the home. *J. Pediatr. Psychol.* 20 23–36. 10.1093/jpepsy/20.1.23 7891237

[B21] Gibson J. J. (1979). *The Ecological Approach to Visual Perception.* Boston, MA: Houghton Miﬄin.

[B22] Gralinski J. H. Kopp C. B. (1993). Everyday rules for behavior: mothers’ requests to young children. *Dev. Psychol.* 29 573–584. 10.1037/0012-1649.29.3.573

[B23] Gray K. Young L. Waytz A. (2012). Mind perception is the essence of morality. *Psychol. Inq.* 23 101–124. 10.1080/1047840X.2012.651387 22754268 PMC3379786

[B24] Grusec J. E. (2011). Socialization processes in the family: social and emotional development. *Annu. Rev. Psychol.* 62 243–269. 10.1146/annurev.psych.121208.131650 20731599

[B25] Grusec J. E. Davidov M. (2010). Integrating different perspectives on socialization theory and research: a domain-specific approach. *Child Dev.* 81 687–709. 10.1111/j.1467-8624.2010.01426.x 20573097

[B26] Grusec J. E. Goodnow J. J. (1994). Impact of parental discipline methods on the child’s internalization of values: a reconceptualization of current points of view. *Dev. Psychol.* 30 4–19. 10.1037/0012-1649.30.1.4

[B27] Harkness S. Super C. M. (2002). “Culture and parenting,” in *Handbook of Parenting: Biology and Ecology of Parenting* Vol. 2 2nd Edn, ed.Bornstein M. H. (Mahwah, NJ: Lawrence Erlbaum Associates Publishers),253–280.

[B28] Hay D. F. (2005). “The beginnings of aggression in infancy,” in *Developmental Origins of Aggression*, eds Tremblay R. E. Hartup W. W. Archer J. (New York, NY: Guilford Press), 107–132.

[B29] Hewlett B. S. (1992). “The parent-child relationship and social-emotional development among Aka pygmies,” in *Parent-child Socialization in Diverse Cultures*, eds Roopnarine J. L. Carter D-B. (Norwood, NJ: Ablex), 223–243.

[B30] Holden G. W. Miller P. C. (1999). Enduring and different: a meta-analysis of the similarity in parents’ child rearing. *Psychol. Bull.* 125 223–254. 10.1037/0033-2909.125.2.223 10087937

[B31] Holm S. (1979). A simple sequentially rejective multiple test procedure. *Scand. J. Stat.* 6 65–70.

[B32] Hox J. (2010). *Multilevel Analysis: Techniques and Applications*, 2nd Edn. New York, NY: Routledge.

[B33] Jagers R. J. Bingham K. Hans S. L. (1996). Socialization and social judgments among inner-city african-american kindergartners. *Child Dev.* 67 140–150. 10.1111/j.1467-8624.1996.tb01725.x

[B34] Kaler S. R. Kopp C. B. (1990). Compliance and comprehension in very young toddlers. *Child Dev.* 61 1997–2003. 10.2307/1130853

[B35] Keenan K. Shaw D. S. (1994). The development of aggression in toddlers: a study of low-income families. *J. Abnorm. Child Psychol.* 22 53–77. 10.1007/BF02169256 8163775

[B36] Killen M. Smetana J. G. (1999). Social interactions in preschool classrooms and the development of young children’s conceptions of the personal. *Child Dev.* 70 486–501. 10.1111/1467-8624.00035

[B37] Kochanska G. (2002). Committed compliance, moral self, and internalization: a mediational model. *Dev. Psychol.* 38 339–351. 10.1037//0012-1649.38.3.339 12005378

[B38] Kochanska G. Aksan N. Joy M. E. (2007). Children’s fearfulness as a moderator of parenting in early socialization: two longitudinal studies. *Dev. Psychol.* 43 222–237. 10.1037/0012-1649.43.1.222 17201521

[B39] Kuczynski L. Kochanska G. Radke-Yarrow M. Girnius-Brown O. (1987). A developmental interpretation of young children’s noncompliance. *Dev. Psychol.* 23 799–806. 10.1037/0012-1649.23.6.799

[B40] LeCuyer-Maus E. A. Houck G. M. (2002). Mother-toddler interaction and the development of self-regulation in a limit-setting context. *J. Pediatr. Nurs.* 17 184–200. 10.1053/jpdn.2002.124112 12094360

[B41] LeVine R. A. (1988). Human parental care: universal goals, cultural strategies, individual behavior. *New Dir. Child Adolesc. Dev.* 1988 3–12. 10.1002/cd.23219884003

[B42] Nucci L. P. Turiel E. (1978). Social interactions and the development of social concepts in preschool children. *Child Dev.* 49 400–407. 10.2307/1128704

[B43] Nucci L. P. Weber E. K. (1995). Social interactions in the home and the development of young children’s conceptions of the personal. *Child Dev.* 66 1438–1452. 10.2307/1131656 7555223

[B44] Power T. G. Parke R. D. (1986). Patterns of early socialization: mother-and father-infant interaction in the home. *Int. J. Behav. Dev.* 9 331–341. 10.1177/016502548600900305

[B45] Reid M. J. O’Leary S. G. Wolff L. S. (1994). Effects of maternal distraction and reprimands on toddlers’ transgressions and negative affect. *J. Abnorm. Child Psychol.* 22 237–245. 10.1007/BF02167902 8064031

[B46] Rijt-Plooij H. H. Plooij F. X. (1993). Distinct periods of mother-infant conflict in normal development: sources of progress and germs of pathology. *J. Child Psychol. Psychiatry* 34 229–245. 10.1111/j.1469-7610.1993.tb00981.x 8444994

[B47] Rogoff B. (2003). *The Cultural Nature of Human Development.* Oxford: Oxford University Press.

[B48] Ross H. S. (1996). Negotiating principles of entitlement in sibling property disputes. *Dev. Psychol.* 32 90–101. 10.1037/0012-1649.32.1.90

[B49] Smetana J. G. (1984). Toddlers’ social interactions regarding moral and conventional transgressions. *Child Dev.* 55 1767–1776. 10.2307/1129924 6705629

[B50] Smetana J. G. (1989). Toddlers’ social interactions in the context of moral and conventional transgressions in the home. *Dev. Psychol.* 25:499 10.1037/0012-1649.25.4.499

[B51] Smetana J. G. (2013). “Moral development: the social domain theory view,” in *The Oxford Handbook of Developmental Psychology: Body and Mind* Vol. 1 ed.Zelazo P. D. (Oxford: Oxford University Press), 832–864.

[B52] Smetana J. G. Braeges J. L. (1990). The development of toddlers’ moral and conventional judgments. *Merrill Palmer Q.* 36 329–346.

[B53] Smetana J. G. Jambon M. Ball C. (2014). “The social domain approach to children’s moral and social judgments,” in *Handbook of Moral Development*, 2nd Edn, eds Killen M. Smetana J. G. (New York, NY: Psychology Press), 23–45.

[B54] Smetana J. G. Kochanska G. Chuang S. (2000). Mothers’ conceptions of everyday rules for young toddlers: a longitudinal investigation. *Merrill Palmer Q.* 46 391–416.

[B55] Smetana J. G. Rote W. M. Jambon M. Tasopoulos-Chan M. Villalobos M. Comer J. (2012). Developmental changes and individual differences in young children’s moral judgments. *Child Dev.* 83 683–696. 10.1111/j.1467-8624.2011.01714.x 22235962

[B56] Srinivasan M. Al-Mughairy S. Kaplan E. Dahl A. (2015). “Preschoolers’ reactions to novel moral and conventional violations,” in *Poster presented at the 9th Biennial Meeting of the Cognitive Development Society*, Columbus, OH.

[B57] Tisak M. S. (1993). Preschool children’s judgments of moral and personal events involving physical harm and property damage. *Merrill Palmer Q.* 39 375–390. 10.2307/23087427

[B58] Tisak M. S. Nucci L. P. Jankowski A. M. (1996). Preschool children’s social interactions involving moral and prudential transgressions: an observational study. *Early Educ. Dev.* 7 137–148. 10.1207/s15566935eed0702_3

[B59] Tremblay R. E. Japel C. Perusse D. McDuff P. Boivin M. Zoccolillo M. (1999). The search for the age of “onset”of physical aggression: Rousseau and Bandura revisited. *Crim. Behav. Ment. Health* 9 8–23. 10.1002/cbm.288

[B60] Turiel E. (1983). *The Development of Social Knowledge: Morality and Convention.* New York, NY: Cambridge University Press.

[B61] Turiel E. (2005). The many faces of parenting. *New Dir. Child Adolesc. Dev.* 108 79–88. 10.1002/cd.130 16121899

[B62] Turiel E. (2008). Thought about actions in social domains: morality, social conventions, and social interactions. *Cogn. Dev.* 23 136–154. 10.1016/j.cogdev.2007.04.001

[B63] Turiel E. (2015). “Moral development,” in *Handbook of Child Psychology and Developmental Science* Vol. 1 ed.Lerner R. M. (New York, NY: John Wiley & Sons, Inc).

[B64] Willems E. P. (1967). Toward an explicit rationale for naturalistic research methods. *Hum. Dev.* 10 138–154. 10.1159/000270581 6062407

[B65] Zahn-Waxler C. Chapman M. (1982). Immediate antecedents of caretakers’ methods of discipline. *Child Psychiatry Hum. Dev.* 12 179–192. 10.1007/BF00706071 7083954

[B66] Zahn-Waxler C. Radke-Yarrow M. Wagner E. Chapman M. (1992). Development of concern for others. *Dev. Psychol.* 28 126–136. 10.1037/0012-1649.28.1.126

